# Teaching Prescribing in the PharmD Curriculum: A Qualitative Analysis

**DOI:** 10.3390/clinpract15120232

**Published:** 2025-12-09

**Authors:** Rachel E. Barenie, Devin Scott, David Rhys Axon, Alina Cernasev

**Affiliations:** 1 College of Pharmacy, University of Tennessee Health Science Center, 881 Madison Avenue, Memphis, TN 38163, USA; 2 Teaching and Learning Center, University of Tennessee Health Science Center, 920 Madison, Suite 424, Memphis, TN 38163, USA; dscott50@uthsc.edu; 3 Division of Pharmacy Practice and Administrative Sciences, James L. Winkle College of Pharmacy, University of Cincinnati, 3255 Eden Avenue, Cincinnati, OH 45267, USA; axondr@ucmail.uc.edu; 4 Department of Clinical Pharmacy and Translational Science, College of Pharmacy, University of Tennessee Health Science Center, 301 South Perimeter Park Drive, Suite 220, Nashville, TN 37211, USA; acernase@uthsc.edu

**Keywords:** medication prescribing, pharmacist, ACPE Standards 2025

## Abstract

**Background:** The scope of practice for pharmacists in the United States (US) is expanding rapidly, with the majority of states allowing pharmacists to prescribe to some degree. Doctor of Pharmacy (PharmD) programs are required to include medication prescribing effective 1 July 2025, ensuring program alignment with modern pharmacy practice. **Objective:** This study aimed to characterize student pharmacists’ beliefs about education on prescribing in the US PharmD program. **Methods:** Focus group discussions (FGDs) were conducted with student pharmacists enrolled in the PharmD curriculum at two different universities in the US. The conceptualization and data collection, guided by Self-Determination Theory, occurred over three months during the 2024 Fall semester. Data analysis was performed using thematic analysis, and themes were identified through inductive and deductive coding. **Results:** Twenty-two student pharmacists participated in three FGDs. Thematic analysis revealed two major themes: (1) essential role of didactic education in the prescribing process and (2) enhancing student preparedness to prescribe through experiential training. These themes uncover student pharmacists’ beliefs that prescribing education is vitally important to the didactic and experiential curriculum, highlighting the need to take a comprehensive approach to incorporate these topics into the PharmD program. **Conclusions:** Teaching medication prescribing in the PharmD didactic curriculum using a state’s scope of practice as a framework for its delivery, with reinforcement in their experiential training, to ensure pharmacy students are practice-ready, may be a preferred approach for delivery. This area remains ripe for further study to determine an evidence-based approach to teaching medication prescribing to pharmacy students.

## 1. Introduction

Pharmacists play a key role in the delivery of healthcare in the United States (US), considering they are one of the most accessible and increasingly more versatile providers due to their expanded scope of practice [1,2]. Pharmacists play a key role in the delivery of healthcare in the United States (US), considering they are one of the most accessible and increasingly more versatile providers. Recently, the Accreditation Council for Pharmacy Education (ACPE) published updated standards (“Standards 2025”), effective 1 July 2025, that require all accredited programs awarding the Doctor of Pharmacy (PharmD) degree to comply with [3]. One key change in Standards 2025 is the requirement to teach medication prescribing in the curriculum. Prior ACPE standards (“Standards 2016”) did not explicitly include this as a required element of the PharmD curriculum [4]. This highlights the recognition of pharmacists’ expanding scope of practice in the US.

In addition to changes in accrediting standards, the pharmacist’s scope of practice is also changing rapidly. The practice of pharmacists is determined by each state, and wide variability exists between states. While teaching this topic will vary depending on what a pharmacist is legally authorized to do in a particular state (i.e., their scope of practice), the fundamental process of medication prescribing remains the same. A pharmacist’s prescriptive authority may be pursuant to a collaborative practice agreement, state protocol, or authorized independently, depending on the state. Ultimately, the pharmacist is—and has been—maintaining patient-provider relationships, conducting patient assessments, developing a therapeutic plan, and implementing that plan to varying degrees. Systematic reviews exist of pharmacists’ prescribing, as well as the views of patients and other key stakeholders on pharmacist prescribing [5]. The research overwhelmingly suggests broad acceptability of this practice. One global systematic literature review on pharmacist prescribing highlights the positive views that other key stakeholders generally hold, with noted benefits including reduced physician workload and ease of patient access [6].

Coupling changes in APCE standards with changes in scope of practice, an important issue is how to effectively educate student pharmacists on prescribing to ensure they are “practice-ready.” Minimal research exists on how to effectively teach prescribing to student pharmacists, even though other health profession degree programs, such as medicine, nursing, and dentistry, have been teaching prescribing for decades. Pharmacy education may utilize existing prescribing frameworks, such as the World Health Organization Guide to Good Prescribing, as a starting point for teaching prescribing in the curriculum [7,8]. Yet, the research that does exist acknowledges the need for further development in this area, which is likely why interventions such as academic detailing have proven to be successful in improving prescribing competency [9,10]. Understanding the provider, student, patient, and educator perspectives is important for teaching prescribing practices. The purpose of this study is to characterize student pharmacists’ beliefs about education on medication prescribing in the PharmD program in the US.

## 2. Methods

### 2.1. Study Design

We conducted a qualitative study using focus group discussions (FGDs) with student pharmacists from two pharmacy colleges in the United States in Fall 2024 (University of Tennessee Health Science Center and University of Arizona). Self-Determination Theory (SDT) served as the theoretical framework for this study, which has been widely applied in healthcare research [11]. SDT consists of three core components: autonomy, competence, and relatedness. SDT can help explain how supportive environments can enhance motivation, job satisfaction, and performance [11]. Pharmacists, for example, are more prone to feel engaged and resilient when they have the freedom to make clinical decisions, feel competent in their roles, and establish supportive relationships with colleagues and patients.

### 2.2. Settings and Participants

After receiving IRB approval from their respective universities (University of Arizona, Study #00004857, approved 11 July 2024, and University of Tennessee Health Science Center, Study #24-10134-XM, approved 16 September 2024), recruitment began. Student pharmacists were recruited through email invitations using listservs that contained emails of all students enrolled at the colleges in all years of the professional program (REB, DRA). Eligible students were those in all four years of the professional pharmacy program at both universities. Emails were sent to all students (University of Arizona, *N* = 526, and University of Tennessee Health Science Center, *N* = 397) in the respective colleges of pharmacy, outlining the study details. Interested participants completed a brief survey indicating their availability for participation. Eligible participants included student pharmacists enrolled in classes at their respective universities, English speakers, and those willing to share their perspectives on prescribing authority. No student pharmacists who completed the IRB consent were eliminated from the study. Upon completing the study, the student pharmacists received a $40 gift card.

### 2.3. Focus Group Guide

The study team developed a semi-structured FGD guide based on the three components of SDT: autonomy, competence, and relatedness [11]. The purpose of this guide was to identify the facilitators and barriers to prescribing authority. It included questions about the benefits of and willingness to prescribe medications, as well as inquiries that aimed to uncover these facilitators and barriers. Some questions were more general, while others were more specific to enhance our understanding of student pharmacists’ perspectives on prescribing. For example, a sample question is, “How comfortable are you with prescribing medications in general? How about chronic diseases such as hypertension and dyslipidemia? And for special populations, such as those with opioid use disorder or those requiring hormonal contraception?” These questions were crafted to explore the competence dimension of SDT. Furthermore, these questions sought to assess student pharmacists’ self-reported confidence in prescribing medications for a range of patient populations and varying levels of complexity [11]. The FGD guide was reviewed and refined after each FGD to improve clarity and include new discussion ideas for future sessions.

### 2.4. Data Collection and Analysis

All FGDs were conducted via online platform (Zoom^®^) over a three-month period in Fall 2024 [12]. We used the COREQ guidelines to guide the data collection and analysis. For example, an experienced qualitative researcher (DS), PhD-trained in qualitative data collection and analysis, who is not a faculty member at either college of pharmacy, led the sessions to ensure that student pharmacists would not feel pressured to provide biased responses (Appendix A) [13]. Student pharmacists selected the date and the time for participation in the FGD, and no observers were present. Student pharmacists were encouraged to share their insights based on their didactic and clinical experiences during the discussions [14]. The facilitator (DS) encouraged independent responses and managed group dynamics to prevent dominant voices from influencing others [13]. Per the consent form, the student pharmacists were encouraged not to disclose the content of their FGD outside. Each student pharmacist participated in only one FGD, which was semi-structured and lasted up to two hours in length [13]. All sessions were audio recorded and transcribed verbatim by a professional transcription company that employs human transcribers [13]. The researchers determined that content saturation was achieved when discussions in the third session began to repeat topics covered in earlier sessions. Thematic analysis is a systematic approach used to extract qualitative themes from data to guide the analysis process [15]. One researcher conducted the analysis and verified the findings, and those findings were peer-reviewed by another team member [13]. We used inductive and conductive analysis and followed the recommended steps outlined by Braun and Clarke to guide the analysis [15]. In the first step, the team familiarized itself with the data by reading and rereading the transcripts [15]. The primary coder, AC, developed the codes, sub-codes, and categories using a deductive approach [15]. Following this, the team met to confirm the coding scheme [15]. The third researcher, REB, joined the team to help resolve any discrepancies that arose during the process. All inconsistencies in coding were discussed and resolved by the entire research team [15]. The team recorded coding definitions, team decisions, and emerging themes in an audit trail to ensure analytic rigor [13]. Dedoose^®^ Software (Version 9.0.17, Los Angeles, CA, USA) was used to organize and manage all qualitative data.

## 3. Results

A total of 22 student pharmacists participated in one of the three FGDs over three months. Three FGDs lasted around 65 min, during which the FGD facilitator wrote field notes. Demographic data were collected and are available in Table 1. Thematic analysis revealed two major themes. In the first theme, student pharmacists emphasized the essential role of the prescribing process in the didactic curriculum. The second theme focused on enhancing student preparedness to prescribe through experiential training, which takes place in the community, institutional, and advanced pharmacy practice experiential components of the curriculum.

### 3.1. Theme 1: Essential Role of Didactic Education in the Prescribing Process

This theme highlights that the current PharmD curriculum is well-equipped educationally and professionally to meet the needs of student pharmacists. However, these participants are also navigating the implications of expanded clinical roles within a complex healthcare system. The FGDs suggest that the pharmacy education curriculum provides a strong foundation for prescribing. The data emphasized the importance of didactic training, clinical rotations, and patient communication in building students’ competence and confidence. In the following excerpt, FG2P4 noted that engaging with clinical guidelines and patient prioritization through coursework mirrors the steps necessary to determine appropriate therapy.

“I think, from a legal standpoint, we’re educated on all things legal in terms of prescribing, what makes a valid prescription, you know, date limits, things of that nature, so I think just having that knowledge alone is very beneficial with regard to prescribing. Definitely from an educational standpoint, just going through our curriculum, we’re taught to manage pretty much every kind of disease state, though that by no means makes us an expert on it. We’re also required to take a pharmacy law class that exposes us to the legality of prescriptions, what is required, who can write them, dates, etc.”

(FG2P4)

Another participant, FG2P6, shared a similar viewpoint regarding academic and rotation preparedness.

“I agree with what FG2P4 said about like our education level classes, like our didactic portion, and then also taking the law class, and then even on from like the ambulatory care aspect, we’re also required to take a medicine rotation. And I think just even going through that lets you—every rotation that you have like shows you the processes behind diagnosing and even prescribing.”

(FG2P6)

A similar sense of confidence and readiness was recognized, particularly in the context of early-career professionals who will naturally depend on mentorship and supervision, as noted in another FGD. This excerpt highlights the broader understanding that readiness to prescribe is not only a product of education but also relies on continued support and practical experience.

“I do think that, as anyone going into a profession, is when they first start out, you have more questions, you might lean on your advisor or those who are in your profession on your practice site…So, under that, I would be comfortable and feel prepared based off of the curriculum and life experiences that I’ve had.”

(FG3P7)

Similarly, another student pharmacist expressed confidence in prescribing due to the didactic education and available resources.

“I do feel somewhat confident, given our education on the resources available, like UpToDate, like Lexicomp or Micromedex.”

(FG1P1)

Despite this level of preparedness, one participant (FG2P5) raised concerns about the evolving identity of pharmacists. This participant expressed worry that expanding prescribing responsibilities might blur the line between pharmacists and physicians, which could deter students who chose pharmacy for its traditional emphasis on medications rather than diagnostics. While many students feel prepared to prescribe, others may see this shift as a challenge to the core values of their chosen profession.

“I do have like more of like a why would people want—it’s just—it’s kind of blurring the lines between going to get your MD versus PharmD. Because if some people go to pharmacy school so that they don’t have to prescribe and they’re just like, okay, we’re just going to do meds and stuff, so then I feel like it could turn people off to pharmacy school if we’re adding on this extra responsibility on top of like all the other stuff that we have to do.”

(FG2P5)

Student pharmacists considered prescribing as a possible part of their role as future pharmacists and identified areas of the PharmD curriculum that could provide the training needed.

### 3.2. Theme 2: Enhancing Student Preparedness to Prescribe Through Experiential Training

This theme reveals a shared belief among participants that hands-on training, mentorship, and ongoing experience are crucial for developing competence in prescribing. This theme highlights that while foundational education is strong, students value and seek practical experience, mentorship, and advanced training to make prescribing a natural and confident part of their professional practice. For instance, FG3P2 emphasizes the value of internship-based learning. FG3P2 expresses confidence that, just as when being supervised when first administering immunizations, the person would receive guidance from practicing pharmacists during the prescribing process. The following excerpt highlights the significance of having supportive preceptors and experiential learning in building comfort with new clinical responsibilities.

“In my current internships, a lot of the training I have received is kind of putting, you know, the rubber to the road. And so I’m certain that as soon as the pharmacists where I am interning start to use their prescriptive authority, that they would take me under their wing and show me the process for doing that.”

(FG3P2)

Another participant suggests incorporating elements of advanced training programs, particularly in ambulatory or primary care, into the PharmD curriculum to enhance preparedness. FG3P1 believes that increased access to structured exposure to advanced clinical roles during pharmacy school would boost students’ confidence and readiness to prescribe.

“I think that some of those maybe like postgrad programs that they have, I know we have one in like ambulatory care, primary care…I think that if those sorts of programs would be integrated into our PharmD curriculum ahead of time, I think that would make me a little more comfortable. Knowing that we already have experienced trained pharmacists in some of these more advanced goals.”

(FG3P1)

Additionally, FG3P5 reinforces the importance of repetition and practical exposure during residency, noting that frequent engagement with prescribing tasks leads to greater fluency and efficiency. FG3P5 clarifies that the need for additional training is not due to a lack of capability but rather a desire to streamline workflows and reduce reliance on external resources such as references.

“…Because, throughout residency, you’ll be exposed to them so often that it—that repetition makes it become second nature in a way. And so that’s why I feel like training comes into—like comes handy.”

(FG3P5)

Student pharmacists proposed integrating prescribing into the existing pharmacy curriculum and providing guidance to preceptors surrounding education during clinical experiences and didactic classes.

## 4. Discussion

This qualitative study, conducted in Fall 2024 with student pharmacists at two colleges of pharmacy in the US, revealed two major themes: (1) essential role of didactic education in the prescribing process and (2) enhancing student preparedness to prescribe through experiential training. Together, these themes highlight student pharmacists’ beliefs that medication prescribing education is vitally important to the didactic and experiential curricula in PharmD programs.

In this study, student pharmacists highlighted the value of hands-on experience to reinforce the decision-making aspect of medication prescribing in practice. Meaningful hands-on experience relates directly to the student’s level of motivation to learn and engage in practice. Prior research has found that participation in learning is higher in learners with more autonomous motivation, linking directly to the competence construct [16]. This experience and learning can be gained during introductory pharmacy practice experiences (IPPEs) and advanced pharmacy practice experiences (APPEs), which are key components of every PharmD program. The core entrustable professional activities (EPAs) for new pharmacy graduates provide a roadmap for the experiential component of PharmD programs, and prescribing may need to be an additional domain not currently included [17,18]. The trajectory of pharmacists’ scope of practice is clearly moving in the direction of independent authority—at least to some degree—and will require that competency. As student pharmacists noted in this study, repetition is key. The extent, however, to which a student may engage in this will vary based on their practice site. In order to ensure continuity and consistency, training for pharmacist preceptors on their expanded scope of practice based on their state law will be key. This will allow students to have practice experiences aligned with the full scope of pharmacy practice. For example, a student may be required to participate in the issuing of a prescription for a certain medication within the pharmacist’s scope of practice in their state. In short, the experiential component of the PharmD curriculum must provide opportunity for repetition and further refinement of these clinical decision-making skills.

Finally, the methods to best teach and reinforce the topic of pharmacist prescribing are limited in pharmacy. A recent systematic review highlighted that there was no consensus on the best way to teach prescribing, but it is clear that teaching on this topic is better than not in ensuring preparedness to prescribe for medical students [19]. Another rapid review found that the World Health Organization Guide to Good Prescribing is a beneficial resource, and teaching this topic in small groups may be effective [20]. More specifically, related to the Self-Determination Theory, literature exists in medical education, suggesting that theory can be easily employed to help better understand effective teaching processes, particularly in medical education, which may be extrapolated to other healthcare professions [21,22]. For example, teachers may engage in “autonomy-supportive teaching behaviors,” allowing students to feel more empowered (competence) and teachers to feel more engaged (relatedness), to facilitate more effective learning, and this method of teaching can be learned and employed by pharmacy educators. This area is ripe for further study, as every PharmD program is required to provide education on prescribing.

### Strengths and Limitations

This qualitative study, grounded in SDT, explores how student pharmacists perceive and develop readiness for prescribing authority. SDT highlights the importance of autonomy, competence, and relatedness in motivating behavior, making it particularly relevant for understanding how educational experiences shape professional identity and clinical confidence. This research design not only identifies strengths and weaknesses in current PharmD curricula but also provides insights into strategies that can enhance self-efficacy and professional development for future prescribing pharmacists. Additionally, the study involved a heterogeneous sample of student pharmacists from two different US geographic areas, which allowed for a broader perspective on prescribing practices. However, the recruitment methods and sample characteristics may limit the generalizability of the findings to other student pharmacists across the US. The sample size aligns with established qualitative research standards, ensuring a depth of richness and robust findings. The in-depth FGD data enabled a compelling thematic analysis, with saturation achieved across all key themes.

## 5. Conclusions

The pharmacists’ scope of practice is expanding, with some state practice acts permitting independent prescriptive authority to a certain degree for pharmacists. Additionally, the ACPE Standards 2025 explicitly require PharmD programs to educate student pharmacists on medication prescribing, effective 1 July 2025 [3]. This study was conducted to identify student pharmacists’ beliefs about medication prescribing education in the PharmD program. The thematic analysis revealed two major themes: (1) essential role of didactic education in the prescribing process and (2) enhancing student preparedness to prescribe through experiential training. Teaching medication prescribing in the PharmD didactic curriculum using a state’s scope of practice as a framework for its delivery, with reinforcement in their experiential training, to ensure pharmacy students are practice-ready, may be a preferred approach for delivery. This area remains ripe for further study to determine an evidence-based approach to teaching medication prescribing to pharmacy students.

## Figures and Tables

**Table 1:** Demographics for Student Pharmacist Participants.

	University of Arizona College of Pharmacy (n = 7)	University of Tennessee College of Pharmacy (n = 15)
Age (years)		
20–24	2	6
25–29	4	6
≥30	1	3
How do you currently describe your gender?		
Female	6	10
Male	1	5
Do you work in a pharmacy?		
Yes	6	13
No	1	2
How many days per month do you work in a pharmacy setting?		
0–2	2	2
3–5	1	7
≥6	4	6
Year in PharmD program		
First year	0	1
Second year	0	4
Third year	5	3
Fourth year	2	7

## Data Availability

The datasets presented in this article are not readily available due to privacy and ethical restrictions.

## Appendix A

**Table A1:** COREQ (COnsolidated criteria for REporting Qualitative research) Checklist.

Topic	Item No.	Guide Questions/Description	Response
**Domain 1: Research team and reflexivity**			
*Personal characteristics*			
Interviewer/facilitator	1	Which author/s conducted the interview or focus group?	Devin Scott
Credentials	2	What were the researcher’s credentials? e.g., PhD, MD	PhD
Occupation	3	What was their occupation at the time of the study?	Assistant Professor and Instructional Consultant
Gender	4	Was the researcher male or female?	Male
Experience and training	5	What experience or training did the researcher have?	PhD. Experienced qualitative researcher trained in qualitative data collection and analysis
Relationship with participants			
Relationship established	6	Was a relationship established prior to study commencement?	No
Participant knowledge of the interviewer	7	What did the participants know about the researcher? e.g., personal goals, reasons for doing the research	Researcher introduced himself and his role in the teaching and learning center. Participants were informed of study goals during the reading of the consent statement.
Interviewer characteristics	8	What characteristics were reported about the inter viewer/facilitator? e.g., Bias, assumptions, reasons and interests in the research topic	None
**Domain 2: Study design**			
*Theoretical framework*			
Methodological orientation and Theory	9	What methodological orientation was stated to underpin the study? e.g., grounded theory, discourse analysis, ethnography, phenomenology, content analysis	Self-Determination Theory
*Participant selection*			
Sampling	10	How were participants selected? e.g., purposive, convenience, consecutive, snowball	Convenience Sample
Method of approach	11	How were participants approached? e.g., face-to-face, telephone, mail, email	Email
Sample size	12	How many participants were in the study?	22
Non-participation	13	How many people refused to participate or dropped out? Reasons?	0
Setting			
Setting of data collection	14	Where was the data collected? e.g., home, clinic, workplace	Virtually, via online platform (Zoom®)
Presence of non- participants	15	Was anyone else present besides the participants and researchers?	No
Description of sample	16	What are the important characteristics of the sample? e.g., demographic data, date	Table 1
*Data collection*			
Interview guide	17	Were questions, prompts, guides provided by the authors? Was it pilot tested?	Sample questions provided: "How comfortable are you with prescribing medications in general? How about chronic diseases such as hypertension and dyslipidemia? And for special populations, such as those with opioid use disorder or those requiring hormonal contraception?"
Repeat interviews	18	Were repeat inter views carried out? If yes, how many?	No
Audio/visual recording	19	Did the research use audio or visual recording to collect the data?	Audio and visual data was collected. Transcription was competed with audio data only.
Field notes	20	Were field notes made during and/or after the inter view or focus group?	Yes
Duration	21	What was the duration of the inter views or focus group?	Up to two hours
Data saturation	22	Was data saturation discussed?	Yes
Transcripts returned	23	Were transcripts returned to participants for comment and/or	No
**Domain 3: analysis and findings**			
*Data analysis*			
Number of data coders	24	How many data coders coded the data?	Two
Description of the coding tree	25	Did authors provide a description of the coding tree?	No
Derivation of themes	26	Were themes identified in advance or derived from the data?	Derived from data, informed by self-determination theory
Software	27	What software, if applicable, was used to manage the data?	Dedoose® Software (Version 9.0.17, Los Angeles, California, USA)
Participant checking	28	Did participants provide feedback on the findings?	No
*Reporting*			
Quotations presented	29	Were participant quotations presented to illustrate the themes/findings? Was each quotation identified? e.g., participant number	Yes
Data and findings consistent	30	Was there consistency between the data presented and the findings?	Yes
Clarity of major themes	31	Were major themes clearly presented in the findings?	Yes
Clarity of minor themes	32	Is there a description of diverse cases or discussion of minor themes?	No

Developed from: Tong A, Sainsbury P, Craig J. Consolidated criteria for reporting qualitative research (COREQ): a 32-item checklist for interviews and focus groups. *Int. J. Qual. Health Care* **2007**, *19*, 349–357 [13].
